# Machines with vision for intraoperative guidance during gastrointestinal cancer surgery

**DOI:** 10.3389/fmed.2022.1025382

**Published:** 2022-09-30

**Authors:** Muhammad Uzair Khalid, Simon Laplante, Amin Madani

**Affiliations:** ^1^Temerty Faculty of Medicine, University of Toronto, Toronto, ON, Canada; ^2^Department of Surgery, University of Toronto, Toronto, ON, Canada; ^3^Surgical Artificial Intelligence Research Academy, University Health Network, Toronto, ON, Canada

**Keywords:** machine learning, computer vision, intraoperative guidance, anatomical landmarking, quality control, task classification

## Introduction

Gastrointestinal (GI) malignancies represent over 26% of all cancers worldwide and a disproportionate 35% of all cancer deaths (1). The most common sites of GI cancers include colorectal (10.0% of all diagnosed cancers), gastric (5.6%), liver (4.7%), esophageal (3.1%), and pancreatic (2.6%) cancers, respectively representing the second (9.4% of all cancer-related deaths), fourth (7.7%), third (8.3%), sixth (5.5%), and seventh (4.7%) most common cause of cancer-related deaths (2). Whereas, the 5-year survival of each of these cancers has been steadily improving over the years (albeit marginally in the case of pancreatic and esophageal cancers), clinical uncertainty has meant that a significant number of these cancers continue to face complications with surgical management (3–7). Indeed, with intraoperative complication rates reaching 40% in some types of gastric cancer resections, patient morbidity and mortality can be significant, especially in oncology-related surgeries (8).

Technology such as artificial intelligence (AI) can potentially play a strong role in improving intraoperative surgical outcomes of gastrointestinal cancers. AI is a field of computer science that uses algorithms to enable machines to mimic higher-order human behaviors like problem-solving and object classification. A subset of AI is machine learning (ML): ML, unlike conventional software, uses inexplicit programming to identify patterns in training datasets, such that when presented with novel data, it is able to make new predictions on that data. A further subset of ML, in turn, is deep learning (DL); DL uses convolutional neural networks (CNNs) that imitate complex human brain pathways using multilayered processing algorithms. CNNs are often black-box (i.e., unexplainable) processes with which machines can learn information and subsequently make decisions in supervised, semi-supervised, and unsupervised settings (9).

At the intersection of ML/AI and image/signal processing is computer vision (CV), a revolutionary new domain that allows machines the ability to understand and interpret visual data. Using CV, algorithms can classify and process pixelated data (i.e., images and videos) *via* point operations, stabilization, and 3D reconstruction; detect and track objects within those images; and perform semantic segmentation (i.e., delineate objects along their boundaries) (10). With much progress in this field over recent years, several applications of CV have been made in diagnostic medicine, including in the determination of diabetic retinopathy from eye images, lung cancer from computed tomography (CT) scans, and skin cancer from images of skin lesions (11–13). Similar progress has also been made in prognostic medicine, where examples include models that use radiomics' analysis from CT imaging studies, back-processing from magnetic resonance imaging series, or digital histopathological slides to predict long-term cardiovascular risk, cancer survival, adverse histopathological status (i.e., advanced tumor-node-metastasis (TNM) staging), or the metastasis of malignancy (14–18).

Despite this, very few surgical applications of CV in the form of intraoperative guidance have made it to patient bedsides. This is because the process of obtaining datasets, annotating, training, testing, validating, and implementing is an extremely complex and resource-intensive process. Indeed, a very recent systematic review looking at the use of machine learning in upper gastrointestinal cancer surgeries found no studies looking at CV or intraoperative guidance (19). In this opinion therefore, we will discuss the current applications of ML/CV in surgery and how they can be used in the intraoperative surgical management of gastrointestinal cancers by providing examples from the literature.

## Intraoperative applications of computer vision in surgery

There are several ways in which computer vision can be used in surgical decision making, especially given that, over the last few decades, there has been a rise in laparoscopic, endoscopic, and robotic surgery. This has allowed researchers in CV to use recorded operative videos for various purposes such as landmark anatomy identification, operative phase recognition, identification of safe and unsafe areas of dissection, coaching, and safety initiatives.

Firstly, CV can be used to identify anatomical landmarks during surgery to aid the surgeon. At our own institution, for instance, we have developed a model (GoNoGoNet) with the ability to replicate the mental model of expert surgeons by recognizing complex anatomical structures without clear boundaries covered by fat and fatty tissues. The model, validated by an external panel of experts, uses laparoscopic cholecystectomy videos as input and overlays Go (with a specificity of 0.97) and No-Go (with a sensitivity of 0.80) zones onto the surgical field (20, 21). Bile duct injuries constitute a major source of avoidable morbidity and mortality in up to 0.7% of laparoscopic cases, and models such as GoNoGoNet have the potential to help guide surgeons by acting akin to an intraoperative GPS (22). The same principle can be applied to oncologic resections. For example, models implemented by two independent groups have attempted to use DL, CNN, and segmentation to identify the total mesorectal excision (TME) plane of dissection during rectal cancer resections (23, 24). This is particularly important given the difficulty of staying in the correct plane of dissection during rectal surgery. Additionally, the correct identification of this plane is key to reducing recurrence, increasing overall survival, and reducing complications such as presacral bleeding and nerve injuries. Despite limited performance in these prototypes, such identification of similar “Go and No-Go zones of dissection” in oncologic rectal surgery shows incredible promise, not only in improving patient outcomes, but also for coaching, setting benchmarks, and education.

Some studies have taken such anatomical and tumor landmarking to the next level by combining intraoperative imaging with preoperative assessments; this is particularly important when trying to identify resection margins and limit the extent of resection during hepatectomy or non-anatomical resections with direct implication on patient outcomes. Examples of these models include surgical navigation systems such as the novel laparoscopic hepatectomy navigation system (LHNS), which fuses preoperative 3D models with indocyanine green (ICG) fluorescence imaging to achieve real-time surgical navigation (25). Systems like LHNS are also able to better recognize liver anatomy and anticipate anatomical changes that occur with retraction as the operation progresses (26).

Secondly, CV can also be used in task classification and quality control checks during surgery. One such example is a group in Strasbourg who was able to create a ML model based in deep neural networks and segmentation, identifying whether the critical view of safety was obtained or not during laparoscopic cholecystectomy with 71.9% accuracy (27). Another example of the use of CV in intraoperative quality control has been in checking anastomotic leaks following cancer resection secondary to inadequate perfusion of the anastomosis. Such leaks can lead to increased recurrence rates, extended hospital stays, and poorer quality of life, eventually causing increased mortality of up to 20% (28). One way to prevent these can be perfusion angiography using ICG. A research group based out of South Korea has, in turn, analyzed angiography images *via* real-time analysis micro-perfusion and CV to predict anastomotic complications with 87% accuracy (29).

Lastly, CV has been shown to help in identification and classification of cancerous lesions at endoscopy. These methods have been trialed in the setting of polyp identification during colonoscopies, showing enhanced ability to detect smaller adenomas (30). Similarly, work has also been done in using AI to aid in the diagnosis of Barrett's esophagus and T1 esophageal cancers with 90 and 85% sensitivity, respectively (31, 32). In its translation to surgical applications, CV could potentially have a role in the identification of tumor invasion, resection margins, or suspicious peritoneal deposits reflective of malignancy at the time of diagnostic laparoscopy.

## Challenges going forward

Many of the examples provided here are in the setting of non-oncological surgery; nevertheless, they are an early proof of concept of the great potential of CV in oncologic surgical care.

Yet, despite the early successes, it must be noted that there are several challenges in developing such ML models in surgery. Firstly, DL approaches are known to be incredibly data hungry, requiring hundreds, if not thousands, of data points to develop a model that has any useful level of accuracy or validity in its predictions (33). Bringing together such amounts of data is challenging, not only in the international collaboration that is required across centers to amalgamate heterogenous data, but also in the time commitment that is needed on behalf of surgeons in curating and annotating operative datasets. As a result, organizations like the Global Surgical AI Collaborative (https://www.surgicalai.org/) are particularly poised to organize and implement DL projects (34). Secondly, the AI algorithms that are developed should not only be computationally-sound, but also designed to address a real unmet clinical need. Doing so requires coordinated work with subject matter experts and other stakeholders, such as cognitive task analyses combined with Delphi consensus, so as to understand the way surgeons think and the milestones they look for while engaged in surgery (35–39).

In conclusion, there are many potential opportunities to apply principles of CV and ML in improving gastrointestinal cancer surgical care. We should aim to make gastrointestinal cancer surgery safer, more effective, and of higher quality by using ML to our advantage in every aspect of care. This will require increased international collaboration and policy development around data storing, sharing, and utilization.

## Author contributions

MK, SL, and AM: idea conceptualization, literature search, manuscript writing–revisions, and final draft. MK: manuscript writing-first draft. All authors contributed to the article and approved the submitted version.

## Conflict of interest

The authors declare that the research was conducted in the absence of any commercial or financial relationships that could be construed as a potential conflict of interest.

## Publisher's note

All claims expressed in this article are solely those of the authors and do not necessarily represent those of their affiliated organizations, or those of the publisher, the editors and the reviewers. Any product that may be evaluated in this article, or claim that may be made by its manufacturer, is not guaranteed or endorsed by the publisher.
